# Re-Evaluating the Contraction Stress Test for Term Fetal Growth Restriction Fetuses: A Retrospective Study †

**DOI:** 10.3390/jcm14165899

**Published:** 2025-08-21

**Authors:** Roie Alter, Hagar Herz, Adiel Cohen, Naama Lessans, Yossef Ezra, Doron Kabiri

**Affiliations:** 1 Department of Obstetrics and Gynecology, Hadassah Ein Kerem Medical Center, Faculty of Medicine, Hebrew University, Jerusalem 9112001, Israel; 2 Faculty of Medicine, Hebrew University, Jerusalem 9112102, Israel

**Keywords:** fetal growth restriction, neonatal outcomes, emergency cesarean delivery, contraction stress test, induction of labor, mode of delivery

## Abstract

**Background:** Fetal growth restriction (FGR) is associated with increased perinatal morbidity and mortality, yet optimal intrapartum management remains debated. The contraction stress test (CST) has been proposed as a tool to assess fetal tolerance to labor, but its prognostic value in FGR pregnancies is unclear. This study aimed to evaluate the utility of CST in predicting perinatal outcomes among term fetuses with FGR and to compare these outcomes with those of small-for-gestational-age (SGA) fetuses. **Methods**: We conducted a retrospective cohort study of term singleton deliveries at a tertiary care center over a two-year period. FGR was defined as birthweight below the 3rd percentile or, prenatally, below the 10th percentile with abnormal Doppler findings. SGA fetuses were defined as birthweights between the 3rd and 10th percentiles. Participants were stratified into the following three groups: (1) FGR with a negative CST result, (2) FGR without CST, and (3) SGA without FGR. The primary outcome was the rate of emergency cesarean delivery. Secondary outcomes included a composite of neonatal adverse events (Apgar score < 7 at 5 min, umbilical cord pH < 7.1, NICU admission, prolonged neonatal hospitalization, intubation, or intraventricular hemorrhage) and a combined metric of neonatal and maternal adverse events. **Results**: A total of 1688 term singleton pregnancies were included in this analysis, comprising 33 cases of FGR with negative CST results, 275 cases of FGR without CST, and 1123 cases classified as SGA. Emergency cesarean delivery rates were comparable between FGR with negative CST (15.2%) and FGR without CST (14.9%), both were significantly higher than in the SGA group (9.7%, *p* = 0.025). Composite neonatal adverse events did not differ significantly between the FGR groups (21.2% vs. 24.7%) but were more frequent than in the SGA group (8.1%, *p* < 0.001). Similarly, the incidence of combined neonatal and maternal adverse events was not different between the FGR groups (30.3% vs. 33.5%) yet exceeded that of the SGA group (15.1%, *p* < 0.001). **Conclusions**: In this cohort, a negative CST performed prior to labor induction did not reduce the risk of adverse maternal or neonatal outcomes in pregnancies complicated by FGR. These findings indicate that routine use of CST may offer limited prognostic benefit in the evaluation of term FGR, highlighting the necessity for further studies to establish evidence-based surveillance and management strategies for this high-risk group.

## 1. Introduction

Fetal growth restriction (FGR) represents a significant obstetric complication defined by a fetus’s failure to achieve its genetically predetermined growth potential. In otherwise healthy pregnancies, the predominant etiology is placental insufficiency; however, additional contributing factors include maternal hypertensive disorders, intrauterine infections, chromosomal or genetic abnormalities, and exposure to harmful substances. FGR is strongly associated with an elevated risk of perinatal morbidity and mortality, particularly among fetuses with birthweights below the 3rd percentile or those exhibiting abnormal umbilical artery Doppler velocimetry. These complications include stillbirth, neonatal intensive care unit (NICU) admission, hypoxic-ischemic encephalopathy, and long-term neurodevelopmental impairment [1,2].

Multiple diagnostic criteria for FGR exist, reflecting the complexity of its identification. Some guidelines rely solely on fetal biometric measurements, such as abdominal circumference (AC) or estimated fetal weight (EFW) below the 3rd percentile, while others incorporate abnormal Doppler findings that indicate placental insufficiency. It is crucial to distinguish FGR from small-for-gestational-age (SGA) status; not all SGA fetuses (defined as birthweight below the 10th percentile for gestational age) are pathologically growth-restricted, as many are constitutionally small but otherwise healthy. This distinction has important implications for clinical management and prognosis.

The optimal delivery mode for pregnancies complicated by FGR remains a subject of ongoing debate. While vaginal delivery is generally preferred for its lower maternal morbidity, FGR fetuses are at heightened risk of intrapartum fetal heart rate decelerations. This vulnerability is reflected by markedly elevated lactate levels in FGR fetuses, relative to their appropriate-for-gestational-age (AGA) peers during labor, indicating diminished resilience to the hypoxic stress of uterine contractions and an increased susceptibility to metabolic acidosis at birth [2]. Consequently, FGR pregnancies with abnormal umbilical artery Doppler velocimetry have reported cesarean delivery rates as high as 75–95% [3]. These elevated rates are likely driven by concerns about fetal compromise during labor, yet they also hinder the ability to rigorously assess outcomes associated with attempted vaginal delivery in this high-risk group.

For SGA fetuses, the emergency cesarean delivery rate is approximately 15%, but prospective studies have shown that SGA babies with abnormal umbilical blood flow experience even higher cesarean rates (17–32%) compared to those with normal Doppler findings (6–9%). Despite these observations, no randomized controlled trials have definitively established the optimal mode of delivery for SGA or FGR fetuses, and FGR alone is not considered an absolute indication for cesarean delivery [4].

Antepartum fetal surveillance aims to identify fetuses at risk of compromise and guide timing and mode of delivery. Traditionally, three main modalities are employed: nonstress test (NST), biophysical profile (BPP), and contraction stress test (CST). The CST is based on the premise that uterine contractions transiently reduce placental blood flow, potentially revealing fetal intolerance to hypoxic stress through late or variable decelerations on fetal heart rate monitoring. A negative CST, defined by the absence of late or significant variable decelerations, has been associated with a very low rate of antepartum stillbirth (0.04%), suggesting a strong negative predictive value for adverse perinatal outcomes [5].

Despite its historical use, the contemporary utility of the CST in FGR management remains uncertain, particularly in the context of modern Doppler-based surveillance. This study was designed to evaluate whether a negative CST performed prior to labor induction in pregnancies complicated by FGR is predictive of improved maternal and neonatal outcomes. We hypothesize that a negative CST result may identify a subset of FGR pregnancies with a lower risk of adverse events, thereby informing clinical decision making and potentially reducing unnecessary interventions.

## 2. Materials and Methods

### 2.1. Study Design and Population

This retrospective cohort study was conducted at two campuses of a tertiary referral hospital, analyzing data from all live, singleton, and SGA deliveries between January 2019 and December 2020. Data were extracted from electronic medical records, and birthweight was recorded immediately after delivery and verified upon neonatal unit admission. SGA was defined as a birthweight below the 10th percentile for gestational age. FGR was determined using the consensus criteria of birthweight below the 3rd percentile or the 10th percentile with abnormal Doppler findings [6]. Exclusion criteria included elective cesarean deliveries, preterm births (<37 weeks of gestation), major congenital anomalies, and out-of-hospital births.

### 2.2. Contraction Stress Test Protocol

The CST was performed using continuous electronic fetal heart rate (FHR) monitoring and external toco-meter. Ten units of oxytocin were diluted in 1000 mL of Hartmann’s solution for a concentration of 10 mU/1 mL. An 18-gauge intravenous line was inserted. The infusion started with an initial rate of 5 mU/min and was increased by 5 mU/min every 15 min. The goal was to achieve at least three uterine contractions within a 10 min window during a 30 min test period. This protocol aligns with established CST procedures, which aim to simulate labor conditions and assess fetal tolerance to the stress of contractions.

### 2.3. CST Interpretation

The CST results were interpreted by the attending senior obstetrician in the delivery room. A negative CST was defined as the absence of late or significant variable decelerations in the FHR tracing, indicating fetal tolerance to contractions. A positive CST was defined by the presence of late decelerations following at least 50% of contractions, reflecting potential fetal compromise due to uteroplacental insufficiency [7]. Cases with equivocal or unsatisfactory results (e.g., inadequate contractions or uninterpretable tracing) were not included in the negative CST group.

### 2.4. Cohort Grouping

Participants were stratified into the following three comparison groups: (1) FGR with negative CST—fetuses diagnosed with FGR who had a negative CST result; (2) FGR without CST—fetuses diagnosed with FGR who did not undergo CST evaluation; and (3) SGA (not FGR)—fetuses with birthweights between the 3rd and 10th percentiles for gestational age and no history of abnormal antepartum Doppler velocimetry.

### 2.5. Outcome Measures

The primary outcome was the rate of emergency cesarean delivery. Secondary outcomes included a composite of neonatal adverse events (Apgar score < 7 at 5 min, umbilical cord pH < 7.1, NICU admission, prolonged neonatal hospitalization, intubation, or intraventricular hemorrhage) and a combined metric of neonatal and maternal adverse events composite of neonatal adverse events, defined as the occurrence of any of the following: Apgar score < 7 at 5 min, umbilical cord blood pH < 7.1, neonatal intensive care unit (NICU) admission, neonatal hospitalization > 4 days, intubation, or intraventricular hemorrhage (IVH).

### 2.6. Statistical Analysis

Categorical variables were analyzed using the chi-square (χ^2^) test or Fisher’s exact test, as appropriate. Quantitative variables were compared between two groups using the independent-samples *t*-test and among three groups using one-way ANOVA. Non-normal variables (e.g., birth weight and hospital stay) were analyzed using the Mann–Whitney U test. Pairwise comparisons were performed using Dunnett’s T3 test, which is appropriate for unequal group sizes and heterogeneous variances, and also adjusts for multiple testing. A post hoc power analysis indicated that the study had 80% power to detect a 25% difference in primary outcomes. All statistical tests were two-tailed, with a significance threshold set at *p* < 0.05. Analyses were performed using IBM SPSS Statistics, version v25.0 (IBM Corp., Armonk, NY, USA).

## 3. Results

**Figure 1:**
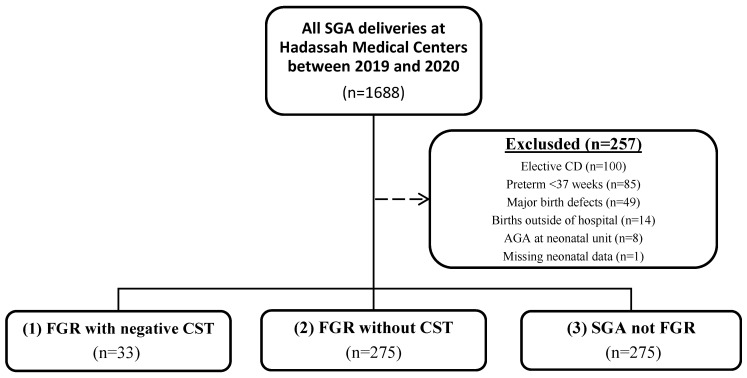
Study Design. SGA—small for gestational age; CD—cesarean delivery; AGA—adequate for gestational age; FGR—fetal growth restriction; CST—contraction stress test.

**Figure 2:**
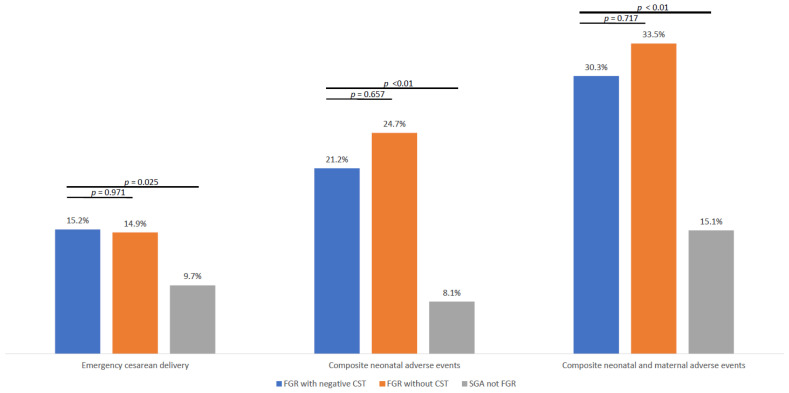
Comparison of emergency cesarean deliveries and composite neonatal and maternal adverse outcomes among FGR with Negative CST, FGR without CST, and the SGA group. FGR: fetal growth restriction; CST: contraction stress test; SGA: small for gestational age.

During the study period, we identified 1688 live, singleton deliveries that were at least SGA in our medical records. After applying exclusion criteria—including elective cesarean delivery (*n* = 100), preterm birth before 37 weeks’ gestation (*n* = 85), major birth defects (*n* = 49), births outside the hospital (*n* = 14), neonates reclassified as AGA at admission (*n* = 8), and cases with missing neonatal data (*n* = 1)—a total of 1,431 cases were included in the final analysis (Figure 1). The cohort was stratified into three groups: (1) FGR with negative CST results (*n* = 33); (2) FGR without CST (*n* = 275); and (3) SGA (*n* = 1123). Baseline maternal and neonatal characteristics for each group are presented in Table 1. The rates of emergency cesarean delivery were comparable between the FGR with negative CST group (15.2%) and the FGR without CST group (14.9%), and both rates were significantly higher than in the SGA group (9.7%, *p* = 0.025; Figure 2).

### 3.1. Composite Neonatal Adverse Events

Composite neonatal adverse events, including Apgar score < 7 at 5 min, umbilical cord pH < 7.1, NICU admission, neonatal hospitalization > 4 days, intubation, or intraventricular hemorrhage, were significantly more frequent in both FGR groups compared to SGA not FGR (21.2% for FGR with negative CST, 24.7% for FGR without CST, and 8.1% for SGA not FGR; *p* < 0.001 for overall comparison). No significant difference was observed between the FGR subgroups (*p* = 0.717).

### 3.2. Composite Neonatal and Maternal Adverse Events

The combined outcome of any composite neonatal adverse event or emergent cesarean delivery was also significantly higher in both FGR groups compared to SGA not FGR (30.3% for FGR with negative CST, 33.5% for FGR without CST, and 15.1% for SGA not FGR; *p* < 0.001 for overall comparison). The difference in composite neonatal adverse events or emergent cesarean delivery between the FGR subgroups was not statistically significant (*p* = 0.717).

## 4. Discussion

The present study evaluated the prognostic utility of the CST prior to labor induction in pregnancies complicated by FGR. Our principal finding is that a negative CST result did not confer any significant reduction in adverse maternal or neonatal outcomes compared to FGR cases managed without CST. Both FGR groups, regardless of CST exposure, exhibited substantially higher rates of emergency cesarean delivery and composite adverse neonatal and maternal outcomes than pregnancies classified as SGA without FGR.

These results prompt a critical reassessment of the CST’s role in contemporary FGR management. While historical data, such as that from Signore et al. [8], have suggested a benefit of CST over the nonstress test (NST) for fetal surveillance in high-risk pregnancies, with reported reductions in neonatal complications and perinatal mortality. Our findings do not support a similar advantage in the modern context. More recent studies, including those by Tanaka et al. and Różańska-Walędziak et al. [9,10], have reported mixed results, with some suggesting that CST may predict certain delivery outcomes or neonatal parameters but without clear evidence of improved overall perinatal outcomes [9]. Notably, our study is the first, to our knowledge, to specifically assess the negative predictive value of CST performed prior to labor induction in FGR pregnancies.

Several mechanisms may account for the observation that negative CST did not reliably confer protection against adverse perinatal outcomes. First, the CST may be relatively insensitive to mild or evolving placental insufficiency, such that only more advanced cases are detected, and less severe compromise escapes detection. Second, when multiple biophysical surveillance methods (such as the nonstress test, biophysical profile, and Doppler studies) are simultaneously employed, the CST’s added predictive value may be minimal due to redundancy. Third, placental function may deteriorate acutely or subacutely after a reassuring test, limiting the temporal window in which the test accurately reflects fetal status. Finally, some adverse outcomes may result from etiologies independent of uteroplacental perfusion, including acute abruption or umbilical cord accidents, further diminishing the assurance that a negative CST provides.

### 4.1. Clinical Implications

While some historical studies suggest benefits, the current study does not demonstrate a reduction in maternal and neonatal complications associated with a negative CST result prior to labor induction in term singleton pregnancies affected by FGR. This finding supports current guideline recommendations [1,4,7,11] that prioritize Doppler-integrated monitoring with NST/BPP over CST in FGR management. This aligns with several models that have tried to predict urgent cesarean deliveries, considering the severity of FGR (EFW < 3rd percentile), cerebral Doppler velocimetry, and the Bishop score [12]. Another cohort study confirmed similar results, with nearly 90% of successful inductions of FGR-affected pregnancies ending in vaginal delivery [13].

### 4.2. Research Implications

The inconclusive role of the CST in FGR management highlights the persistent unanswered question regarding the optimal management of FGR-affected fetuses. However, it is possible that using a combined model that considers the severity of the growth restriction (i.e., EFW < 3rd percentile), placental function represented by Doppler flows (cerebroplacental ratio and uterine artery velocimetry), and maternal cervical conditions represented by the Bishop score, instead of a single predictor, will provide better prediction of the mode of delivery.

#### Future Research Directions

Advancing the management of FGR will require several research steps. Prospective, multicenter trials should assess the utility of the CST across different FGR severity profiles and compare CST-based strategies with modern, Doppler-integrated surveillance approaches using standardized outcomes. There is also a need to develop and validate integrated risk stratification models that combine CST findings with Doppler indices, cervical status, and relevant maternal characteristics to improve individualized risk assessment. Establishing consensus guidelines for CST indications, administration, and interpretation will allow for reliable comparisons across studies and clinical settings. Finally, systematic follow-up of long-term neurodevelopmental and health outcomes is essential to determine if differences in surveillance strategies translate into meaningful benefits, ultimately guiding future practice recommendations.

### 4.3. Strengths and Limitations

The strengths of this study include its large, well-characterized cohort and the use of composite outcome measures that capture both neonatal and maternal morbidity. By directly comparing FGR pregnancies with and without CST, as well as with an SGA reference group, the analysis offers a nuanced assessment of the CST’s incremental clinical value.

#### Limitations

Several limitations must be acknowledged in our study. First, the retrospective design introduces inherent biases and limitations, which restrict our ability to control for confounding variables that may influence clinical decision-making and outcomes. Second, the relatively small size of our CST subgroup (*n* = 33) may limit the statistical power to detect clinically meaningful differences and affect the generalizability of our findings. Third, our analysis focused on short-term perinatal outcomes and lacked long-term neonatal developmental data, which would provide more comprehensive insight into the clinical utility of CST. Finally, selection bias may have influenced which FGR cases underwent CST versus standard monitoring, as the decision to perform CST was at the discretion of the attending physician and may have been influenced by unmeasured clinical factors or perceived risk levels

### 4.4. Conclusions

Our findings suggest that the routine use of contraction stress testing (CST) prior to labor induction in pregnancies complicated by fetal growth restriction (FGR) does not reduce the risk of adverse maternal or neonatal outcomes, and may not provide additional prognostic information beyond established clinical and Doppler-based criteria. These results support current guideline recommendations to prioritize Doppler-driven surveillance protocols over routine CST in term FGR management.

Given the inherent limitations of retrospective analyses and the ongoing evolution of fetal surveillance strategies, our study highlights the need for well-designed, prospective research to clarify the optimal role of CST in this setting. Until such data are available, clinicians should carefully weigh the utility of CST against alternative surveillance modalities and adopt individualized, multifactorial approaches tailored to each FGR pregnancy.

## Figures and Tables

**Table 1:** Comparison of maternal delivery and neonatal characteristics between study groups.

	FGR with Negative CST n = 33 (2.3%)	FGR Without CST n = 275 (19.2%)	SGA Not FGR n = 1123 (78.5%)	*p*-Value
Maternal				
Age, years	28.55 ± 5.21 [21–40]	29.55 ± 5.81 [18–44]	-	0.342
BMI, kg/m^2^	27.47 ± 5.63	24.95 ± 4.63	-	0.004
Parity	2.27 ± 2.02	2.51 ± 1.96	-	0.521
Age, years	28.55 ± 5.21 [21–40]	29.55 ± 5.81 [18–44]	28.09 ± 5.36 [18–46]	0.998
BMI, kg/m^2^	27.47 ± 5.63	24.95 ± 4.63	25.29 ± 4.62	0.023
Parity	2.27 ± 2.02	2.51 ± 1.96	2.31 ± 1.74	0.249
Labor and Delivery				
Gestational Age, weeks	37.85 ± 0.83 [37–40]	38.89 ± 1.24 [37–42]	-	<0.001
Induction of Labor	25 (89.3)	90 (38.3)	-	<0.001
Vaginal Delivery	22 (66.7)	200 (72.7)	-	0.464
Cesarean Delivery	5 (15.2)	41 (14.9)	-	0.971
Gestational Age, weeks	37.85 ± 0.83 [37–40]	38.89 ± 1.24 [37–42]	39.38 ± 1.15 [37-42]	<0.001
Induction of Labor	25 (89.3)	90 (38.3)	255 (24.9)	<0.001
Vaginal Delivery	22 (66.7)	200 (72.7)	846 (75.4)	0.085
Cesarean Delivery	5 (15.2)	41 (14.9)	109 (9.7)	0.025
Neonatal				
Male	14 (42.4)	115 (41.8)	-	0.947
Birthweight, gram	2294 ± 190 [1834–2570]	2301 ± 197 [1638–2700]	-	0.848
5 min Apgar < 7	0	3 (1.1)	-	1
Umbilical Artery pH	7.27 ± 0.10 [6.97–7.38]	7.27 ± 0.09 [7.00–7.48]	-	0.957
pH < 7.2	1 (7.1)	31 (18.9)	-	0.470
pH < 7.1	1 (7.1)	9 (5.5)	-	0.569
Admission Length, days	4.48 ± 3.193 [2–18]	4.53 ± 4.318 [1–42]	-	0.956
>4 days	6 (18.2)	64 (23.3)	-	0.510
>7 days	2 (6.1)	25 (9.1)	-	0.752
NICU Admissions	2 (6.1)	22 (8.0)	-	1
IVH	0	1 (0.4)	-	1
Mechanical Ventilation				0.634
NIPPV	3 (9.1)	18 (6.5)	-	
Intubation	0	3 (1.1)	-	
Male	14 (42.4)	115 (41.8)	562 (50)	0.040
Birthweight, gram	2294 ± 190 [1834–2570]	2301 ± 197 [1638–2700]	2651 ± 170 [2180–3300]	<0.001
-min Apgar < 7	0	3 (1.1)	5 (0.4)	0.333
Umbilical Artery pH	7.27 ± 0.10 [6.97–7.38]	7.27 ± 0.09 [7.00–7.48]	7.28 ± 0.08 [7.02–7.46]	0.383
pH < 7.2	1 (7.1)	31 (18.9)	115 (17.7)	0.634
pH < 7.1	1 (7.1)	9 (5.5)	16 (2.5)	0.054
Admission Length, days	4.48 ± 3.193 [2–18]	4.53 ± 4.318 [1–42]	2.80 ± 1.543 [0–26]	<0.001
>4 days	6 (18.2)	64 (23.3)	71 (6.3)	<0.001
>7 days	2 (6.1)	25 (9.1)	15 (1.3)	<0.001
NICU Admissions	2 (6.1)	22 (8.0)	15 (1.3)	<0.001
IVH	0	1 (0.4)	1 (0.1)	0.384
Mechanical Ventilation				0.089
NIPPV	3 (9.1)	18 (6.5)	47 (4.2)	
Intubation	0	3 (1.1)	4 (0.4)	
Composite Outcome				
Composite Neonatal Adverse Events	7 (21.2%)	68 (24.7%)	-	0.657
Composite Neonatal and Maternal Adverse Events	10 (30.3%)	92 (33.5%)	-	0.717
Composite Neonatal Adverse Events	7 (21.2%)	68 (24.7%)	91 (8.1%)	<0.001
Composite Neonatal and Maternal Adverse Events	10 (30.3%)	92 (33.5%)	170 (15.1%)	<0.001

Shaded cells represent only the FGR groups (1 vs. 2). Continuous variables are presented as the mean ± standard deviation (range), and numerical values are presented as N (%). NICU: neonatal intensive care unit; IVH: intraventricular hemorrhage; NIPPV: noninvasive positive pressure ventilation.

## Data Availability

The datasets generated and analyzed during the current study are not publicly available but are available from the corresponding author upon reasonable request.

## Abbreviations

**CST**: Contraction Stress Test; **FGR**: Fetal Growth Restriction; **SGA**: Small for Gestational Age; **AC**: Abdominal Circumference; **EFW**: Estimated Fetal Weight; **NST**: Non-Stress Test; **BPP**: Biophysical Profile; **FHR**: Fetal Heart Rate; **NICU**: Neonatal Intensive Care Unit; **IVH**: Intraventricular Hemorrhage; **IRB**: Institutional Review Board.
